# Comparison of bougie-guided insertion of Proseal™ laryngeal mask airway with digital technique in adults

**DOI:** 10.4103/0019-5049.60494

**Published:** 2010

**Authors:** Anand Kuppusamy, Naheed Azhar

**Affiliations:** Department of Anaesthesiology, Madras Medical College, Chennai - 600 003, Tamilnadu, India; 1 Department of Anaesthesiology, Govt Dharmapuri Medical College, Dharmapuri - 636705. Tamilnadu, India

**Keywords:** Anaesthesia, airway, equipment, laryngeal masks, technique, oropharyngeal seal pressure

## Abstract

The Proseal™ laryngeal mask airway (PLMA™, Laryngeal Mask Company, UK) was designed to improve ventilatory characteristics and offer protection against regurgitation and gastric insufflation. The PLMA is a modified laryngeal mask airway with large ventral cuff, dorsal cuff and a drain tube. These modifications improve seal around glottis and enable better ventilatory characteristics. The drain tube prevents gastric distension and offers protection against aspiration. There were occasional problems, like failed insertion and inadequate ventilation, in placing PLMA™ using the classical digital technique. To overcome these problems, newer placement techniques like thumb insertion technique, introducer tool placement and gum elastic bougie (GEB)-aided placement were devised. We compared classical digital placement of PLMA™ with gum elastic bougie-aided technique in 60 anaesthetised adult patients (with 30 patients in each group) with respect to number of attempts to successful placement, effective airway time, airway trauma during insertion, postoperative airway morbidity and haemodynamic response to insertion. The number of attempts to successful placement, airway trauma during insertion and haemodynamic response to insertion were comparable among the two groups, while effective airway time and oropharyngeal leak pressure were significantly higher in bougie- guided insertion of PLMA. Postoperatively, sore throat was more frequent with digital technique while dysphagia was more frequent with bougie guided technique. Hence gum elastic bougie guided, laryngoscope aided insertion of PLMA is an excellent alternate to classical digital technique.

## INTRODUCTION

Laryngeal Mask Airway (LMA) combines the advantages of a non-invasive face mask and the more invasive endotracheal tube. Originally, LMA was recommended as a better alternative to the face mask. However, ever since its development, the LMA has challenged the assumption that tracheal intubation is the only acceptable way to maintain a clear airway and provide positive pressure ventilation. Since its commercial introduction in 1988, it has been used in over 200 million routine and emergency procedures. Though LMA provides all the above advantages, the risk of gastric distension, pulmonary aspiration of gastric contents and fear of inadequate ventilation acts as a deterrent to the widespread use of LMA.

To overcome the above complications, Dr. Archie Brain designed the Proseal Laryngeal Mask Airway (PLMA)™ in 2000, with modified cuff to improve seal around glottis. The main aim of Drain Tube (DT) is to enhance scope and safety of the device, particularly when used with positive pressure ventilation.[1–3] Adult studies have shown that compared to classic laryngeal mask airway, the PLMA forms a better seal with both respiratory and gastrointestinal tract and provides easy access to the gastrointestinal tract.[4,5] The PLMA (when placed by the classical digital technique) also poses occasional problems during placement, leading to risk of inadequate ventilation.[6,7] To overcome these problems, newer placement techniques like the thumb placement, Introducer tool placement and GEB (Gum Elastic Bougie)-aided placement[6,8–10] were described. All these new techniques touted higher success rates and better placement of the PLMA.

With this background, this study was conceptualised to compare the classical digital placement technique of the PLMA with the Gum Elastic Bougie-aided placement technique.

## METHODS

This study was a randomised, prospective, comparative study. After obtaining Institutional Ethical committee clearance and patients' written informed consent, the study was carried out at Madras Medical College and Government General Hospital, Chennai. The study was conducted on 60 adult patients of either sex, in the age group of 18-80 years, belonging to ASA I and II posted for elective minor surgeries. 0 Adults of either sex assessed under ASA PS I/II with Modified Mallampati Score I/II were included in the study. Patients with increased risk of aspiration (like hiatus hernia, gastro oesophageal reflux disease (GERD), obesity, pregnancy) and patients with anticipated difficult airway (like inter incisor distance<2cm, Modified Mallampatti Score 3 and 4) were excluded from this study.

### Study method

Patients were randomised into two groups using sealed envelope technique.

- Group D - PLMA insertion by digital technique
- Group G - PLMA insertion by GEB-guided technique

All patients were kept nil per oral overnight. They were given aspiration prophylaxis with Inj. Ranitidine 50 mg IV and Inj. Metoclopromide 10 mg IV one hour before surgery. Patients were premedicated with Inj. Glycopyrrolate 0.2 mg IV one hour before surgery. After the placement of standard minimum monitoring devices [ECG, SpO_2,_ NIBP, and Capnography] and preoxygenation, all the patients were induced with Inj.Fentanyl 2 mcg/kg IV, Inj.Lignocaine 1.5 mg/kg, Inj.Propofol 3 mg/kg I.V. PLMA was inserted by digital/bougie-guided technique according to the study group. The investigators were anaesthesiology residents who had performed at least 20 PLMA insertions prior to study.

### Group D - Digital technique

PLMA was selected as per body weight chart and inserted using index finger as recommended by manufacturer.

### Group G - Gum elastic bougie guided insertion

The Proseal LMA drain tube was primed with well lubricated 16F GEB with straight end protruding 30 cm beyond drain tube. Under laryngoscopic guidance, distal portion of GEB was placed 5 to 10 cm into the oesophagus. The laryngoscope was removed and PLMA was inserted using digital technique, while an assistant stabilized the proximal end of the bougie. The bougie was removed while PLMA was held in position.[6,8–10] All insertions were performed in sniffing position with cuff fully deflated and using midline approach.

Three attempts were allowed before insertion was considered a failure. Criteria for failed insertion include:

- failed passage into pharynx
- malposition as detected by air leak over oropharynx (listening over mouth)/stomach (auscultation over epigastrium)/drain tube (placing lubricant over proximal drain tube) and negative suprasternal notch tap test.
- ineffective ventilation (exhaled tidal volume TVe <8 ml/Kg and ET CO_2_>45 mm Hg

The time between picking up laryngoscope/PLMA and successful placement was recorded. When insertion was successful, intracuff pressure was set at 60 cm H_2_O using cuff pressure monitor (Endotest™). Any episode of hypoxia (SpO_2_<90%) or other adverse events were noted. In the event of a failed insertion of PLMA after three attempts, patient was intubated with an endotracheal tube and surgery was allowed to proceed. Oropharyngeal leak pressure was measured as the pressure at which audible leak is heard at a constant flow of 6L/min with Adjustable Pressure Leak valve kept closed. Pulse rate, blood pressure, (systolic, diastolic, mean arterial pressure) were recorded prior to insertion and one, three, five, ten minute intervals after insertion, Anaesthesia was maintained with N_2_O 2 litres/minute and O_2_ 1 litre/minute, Isoflurane 0.8-1.5% and patient was allowed to breath spontaneously. Exhaled tidal volume (TV_e_) of at least 8 ml/kg and ETCO_2_<45 mm Hg was maintained. At the end of procedure, PLMA was removed after recovery criteria were met. Any visible blood staining on PLMA, laryngoscope, bougie was noted down. Mouth, lips, tongue were inspected for any evidence of trauma.

Patients were interviewed 18-24 hours postoperatively regarding sore throat (constant pain even without swallowing), dysphonia (difficulty or pain on speaking), dysphagia (difficulty or pain on swallowing). The intraoperative data was collected by unblinded trained observers while postoperative data was collected by two blinded trained observers.

### Statistical analysis

The sample size for the study was based on a pilot study on 10 patients. The outcome of pilot study indicated that a sample size of 30 in each group would give enough power of more than 85%. However, results of the pilot study are not included in the results of main study. The results were analysed statistically using student t test and chi square test, wherever appropriate. Differences were considered to be statistically significant when *P* value was<0.05.

## RESULTS

Both the groups are statistically comparable with respect to demographic variables like age, sex and weight. The effective airway time for GEB-guided insertion of PLMA was longer than that of digital technique (36.87±11.2 seconds vs. 22.32±12.09 seconds). GEB-guided PLMA insertion was successful in 96.7% patients in first attempt, while only one patient required second attempt. PLMA insertion with digital technique was successful in 86.7% patients in first attempt, while 10% of insertions required second attempt. PLMA could not be inserted in one patient with digital technique even after three attempts. However, the difference in success rates between both groups is not statistically significant. Clinically, GEB-guided PLMA insertion seemed to be associated with decreased incidence of malpositioning, though statistical analysis did not reveal significant difference. Oropharyngeal leak pressure in digital technique was 23.13±3.69 mm Hg while that with GEB-guided technique was 30.63±4.71 mm Hg (*P*=0.001, significant). The incidence of blood staining on PLMA was same in both the groups (16.6%). There was no statistically significant difference in incidence of airway trauma between both the groups. Sore throat occurred in 10% of patients in digital technique, while it was not noted in GEB technique. But the difference is not statistically significant Table 1.

**Table 1:** Comparative data for digital technique and GEB technique

Variables	Group D	Group G	*P* value
No. of patients	30	30	
Demographic data			
Age (years)	38.8	43.6	NS
	(11.04)	(12.6)	NS
Male: Female (n)	24:6	20:10	NS
Weight (Kg)	52.6	56.8	
	(15.4)	(16.4)	
Effective airway time	36.877	22.327	0.001
(Sec)	(11.21)	(12.090)	Significant
Number of attempts (1/2/3)	26/3/0	29/1/0	NS
Malposition			
FPP (Y/N)	2/28	1/29	NS
AL-O (Y/N)	2/28	1/29	NS
AL-G- (Y/N)	2/28	1/29	NS
AL-D (Y/N)	2/28	1/29	NS
SSN-TT (Y/N)	4/26	1/29	NS
IV (Y/N)	4/26	1/29	NS
OP leak pressure (mmHg)	23.13 (3.693)	30.63	0.001
		(4.716)	Significant
Visible blood staining			
Laryngoscope (Y/N)	NA	0/30	NS
PLMA (Y/N)	5/25	5/25	NS
GEB (Y/N)	NA	1/29	NS
Airway trauma during insertion	0/30	0/30	NS
Tongue (Y/N)	1/29	1/29	NS
Lips (Y/N)	0/30	2/25	NS
Mouth (Y/N)			
Postop airway morbidity			
Sorethroat (Y/N)	3/27	0/30	NS
Dysphonia (Y/N)	0/30	0/30	NS
Dysphagia (Y/N)	0/30	5/25	0.02
			Significant.

Values are given as mean (s.d) and number (n), GEB: Gum elastic bougie

Dysphagia occurred in 16.7% of patients in GEB group while it was not observed in digital technique (*P*=0.02, Chi square test, significant). There is no significant difference in haemodynamic response to PLMA insertion between both the techniques Table 2.

**Table 2:** Haemodynamics

	Group	n	Mean	Std. deviation	Student t-test	
Heart rate						
Preinsertion	D	30	83.37	14.583	t=1.07	*P*=0.29
	G	30	87.70	16.670		
1 min	D	30	79.93	10.754	t=0.84	*P*=0.40
	G	30	82.50	12.757		
3 min	D	30	78.87	11.016	t=0.42	*P*=0.67
	G	30	80.10	11.436		
5 min	D	30	78.80	11.845	t=0.15	*P*=0.88
	G	30	79.23	10.566		
10 min	D	30	77.93	10.793	t=0.07	*P*=0.94
	G	30	78.13	10.817		

Systolic blood pressure							
Preinsertion	D	30	130.40	15.144	t=0.97	*P*=0.33
	G	30	134.33	16.076		
1 min	D	30	99.33	13.639	t=0.35	*P*=0.72
	G	30	100.67	15.363		
3 min	D	30	97.13	9.804	t=1.52	*P*=0.13
	G	30	102.00	14.532		
5 min	D	30	102.13	11.252	t=1.89	*P*=0.07
	G	30	108.63	15.082		
10 min	D	30	110.13	10.849	t=0.94	*P*=0.35
	G	30	113.23	14.318		

Diastolic blood pressure						
Preinsertion	D	30	81.80	9.690	t=1.01	*P*=0.31
	G	30	84.43	10.318		
1 min	D	30	64.70	9.374	t=1.74	*P*=0.09
	G	30	69.20	10.584		
3 min	D	30	64.87	7.655	t=1.04	*P*=0.30
	G	30	67.50	11.524		
5 min	D	30	68.37	7.703	t=1.65	*P*=0.10
	G	30	72.30	10.475		
10 min	D	30	73.97	8.834	t=0.51	*P*=0.61
	G	30	75.23	10.451		

Mean arterial pressure						
Preinsertion	D	30	97.97	11.044	t=0.87	*P*=0.39
	G	30	100.57	12.193		
1 min	D	30	76.03	10.404	t=1.25	*P*=0.22
	G	30	79.57	11.533		
3 min	D	30	75.33	7.373	t=1.46	*P*=0.15
	G	30	79.07	11.925		
5 min	D	30	79.23	8.361	t=1.95	*P*=0.06
	G	30	84.43	11.866		
10 min	D	30	85.67	9.452	t=0.77	*P*=0.45
	G	30	87.70	11.033		

## DISCUSSION

The first attempt success rate was higher with GEB technique than digital technique. Several studies conducted on adult and paediatric patients confirm this finding.[8,9–13] The higher first attempt success rate with GEB technique is due to the fact that the incidence of distal cuff folding over is reduced when PLMA is primed with GEB. The effective airway time was longer with GEB-guided insertion than digital technique in our study. This is in concurrence with the studies conducted in adult and paediatric patients.[9,12,13] Effective airway time was longer with GEB-guided technique because of increased time needed for laryngoscopy and GEB placement.

The commonest cause of failed insertion in both the groups was malposition of PLMA, as detected by negative suprasternal notch tap test.[7] The incidence of malposition was higher with digital technique. However, in other studies, failed passage into pharynx and glottic impaction were the commonest cause of malposition.[10,12]

We found that oropharyngeal leak pressure was higher with GEB technique (30.6 cm H_2_O), compared to digital technique (23 cm H_2_O). This is in concurrence with other studies which shows higher oropharyngeal leak pressure with GEB technique.[8,10,11]

Our study showed no difference in the incidence of visible blood staining on PLMA with both the techniques. This is in concurrence with other studies which shows similar incidence of blood staining on PLMA with both techniques.[9,10] Only one patient had trauma on lips in both groups; but no patient had trauma over mouth/tongue in both groups in our study. However, in 2005, Lopez-Gil *et al.* found higher incidence of trauma on mouth and lips in GEB group.[12] This could be due to the fact that their study was on children, while our study was on adults.

Sore throat was more frequent in digital technique while dysphagia was more frequent with GEB technique. The higher incidence of dysphagia associated with GEB technique can be attributed to placement of gum elastic bougie in oesophagus during PLMA insertion by GEB technique. The incidence of dysphagia with GEB technique and incidence of sore throat with digital technique[8,14] is comparable to other studies. We found no significant difference in haemodynamic response to PLMA insertion by digital or GEB technique. This finding is in concurrence with other studies.[8,10,12,14]

Our study has a few limitations. Fibre optic grading of PLMA placement was not done and intraoperative data was collected by unblinded observers. However, postoperative data was collected by blinded observers.

## CONCLUSION

We conclude that the GEB-guided, laryngoscope aided insertion of PLMA is an excellent alternative technique to digital technique in adults. Though bougie-guided insertions of PLMA took longer time, they helped achieve higher oropharyngeal leak pressure and less number of failed insertions.
